# Learning artificial number symbols with ordinal and magnitude information

**DOI:** 10.1098/rsos.220840

**Published:** 2023-06-07

**Authors:** Hanna Weiers, Matthew Inglis, Camilla Gilmore

**Affiliations:** Centre for Mathematical Cognition, Loughborough University, Loughborough LE11 3TU, UK

**Keywords:** symbol-grounding problem, artificial symbol learning, magnitude vs ordinality, symbolic comparison, order judgement, cross-modal comparison

## Abstract

The question of how numerical symbols gain semantic meaning is a key focus of mathematical cognition research. Some have suggested that symbols gain meaning from magnitude information, by being mapped onto the approximate number system, whereas others have suggested symbols gain meaning from their ordinal relations to other symbols. Here we used an artificial symbol learning paradigm to investigate the effects of magnitude and ordinal information on number symbol learning. Across two experiments, we found that after either magnitude or ordinal training, adults successfully learned novel symbols and were able to infer their ordinal and magnitude meanings. Furthermore, adults were able to make relatively accurate judgements about, and map between, the novel symbols and non-symbolic quantities (dot arrays). Although both ordinal and magnitude training was sufficient to attach meaning to the symbols, we found beneficial effects on the ability to learn and make numerical judgements about novel symbols when combining small amounts of magnitude information for a symbol subset with ordinal information about the whole set. These results suggest that a combination of magnitude and ordinal information is a plausible account of the symbol learning process.

## 1. Introduction

Numbers are both important and ubiquitous. They are needed when we look at house numbers or prices in the supermarket, or when we estimate how long a walk will take. How humans represent and process numbers has therefore been extensively investigated. One key question concerns how numerical symbols, such as Arabic digits or number words, gain semantic meaning. This is often referred to as the symbol-grounding problem [1]. Although several solutions to the symbol-grounding problem have been proposed, none has gained widespread acceptance. It is therefore important to investigate how numerical symbols can be learned and what role the type of information provided about these symbols plays. Here we report two studies about adults' learning of artificial symbols, which explore the types of information that can give meaning to numerical symbols. We first outline different accounts for the symbol-grounding problem, before considering how artificial learning experiments may contribute to this debate.

Semantic meaning for number symbols can come from magnitude information (i.e. the quantity of items a symbol represents) or ordinal information (i.e. the position of the symbol in a sequence). There is evidence that both these types of information play a role in adults’ use of number symbols. For example, adults' processing of number symbols is related to both judgements of magnitude (e.g. [2]) and order (e.g. [3]) and that these have separate influences [4]. These studies demonstrate that both magnitude and ordinal information may be involved in adults' use of number symbols, but they don't tell us about number symbol learning. Alternative proposals exist regarding the role of magnitude and ordinal information when learning number symbols.

One prominent suggestion is that symbols and number words gain meaning by being mapped onto a pre-existing, non-symbolic representation of number magnitude (e.g. [1,5]). Indeed, research has shown that humans have an intuitive understanding of quantities, or a ‘number sense’. The approximate number system (ANS), also known as analogue magnitude system, has been proposed to explain how numerical symbols gain semantic meaning.

The ANS is present throughout the lifespan [6] and allows the automatic processing, approximate estimation and manipulation of representations of numerosities [1,7–9]. Although the ANS does not represent numbers exactly and does not allow successor relations (i.e. that each number is exactly one more than the previous one) [10], the ANS is considered by some to be a basis for number processing and further mathematical abilities (e.g. [6,11,12]). One of the main signatures of the ANS is ratio effects. An individual's ANS acuity is commonly measured using non-symbolic comparison tasks in which participants are required to compare two non-symbolic quantities (e.g. dot arrays) and judge which one is larger in numerosity. Performance is often described in relation to the ratio (or the distance) on a given comparison. It is commonly found that reaction times (RTs) increase and accuracy decreases as the ratio between the to-be-compared numerosities approaches 1. The closer the ratio is to 1, the more difficult it is to differentiate two numerosities, for example, individuals are less accurate and slower to respond [13,14] when comparing 9 versus 10 (0.9 ratio) than 5 versus 10 (0.5 ratio). The ratio effect is well established and has been demonstrated in behavioural as well as neuroimaging data (e.g. [4,15–20]).

Evidence for the role of the ANS in numerical symbol processing comes from studies showing that adult performance on symbolic comparison tasks (i.e. choosing the larger of two Arabic digits) mirrors adult performance on non-symbolic comparison tasks. That is, performance on both symbolic and non-symbolic comparison tasks is characterized by similar ratio effects. This has been taken to suggest that the same underlying mechanism is involved in both, and symbolic numbers take on the signatures of non-symbolic quantities (e.g. [1,5,21]). Furthermore, there is a consistent, albeit weak, relationship between performance on non-symbolic comparison tasks and mathematical tasks [2].

However, other studies have questioned the close association between symbolic and non-symbolic numerical processing. Some studies investigating correlations between symbolic and non-symbolic comparison abilities have not only failed to find significant correlations between these skills, but have also found that non-symbolic comparison skills did not predict future symbolic skills [22,23]. Lyons *et al*. [24] highlighted that if non-symbolic quantities are the basis for the acquisition of symbolic number meaning, and if symbolic representations of number were indeed mapped onto non-symbolic ones, then performance on a cross-modal comparison task (i.e. judging whether an Arabic digit or dot array is larger in numerosity) should not be significantly worse than a comparison within just one of the modalities (judging which of two dot arrays or Arabic digits is larger in numerosity). Nevertheless, they found that the cross-modal comparison between symbolic and non-symbolic formats was more difficult than comparing two non-symbolic stimuli. Thus, they did not support the ANS mapping account, but instead suggested that the relation between two symbols overshadows that of a symbol with its quantity.

Given this evidence of a disconnect between symbolic and non-symbolic representations, an alternative solution to the symbol-grounding problem proposes that numerical symbols do not automatically activate the non-symbolic representation of the quantity they represent, but instead that numerical symbols gain meaning when considered in terms of their relative position or order with respect to other symbols [24–26]. Indeed, an increasing body of research indicates that ordinal information is important for symbolic representations of number [19] and that non-verbal, non-symbolic numerosities are increasingly understood in symbolic terms [27–30].

Numerical order processing is commonly investigated with order judgement tasks. These involve judging whether a presented numerical triplet (or pair) is in ascending order. Performance on these tasks is often characterized by a reverse distance effect (RDE), which has been replicated across numerous different studies (e.g. [4,19,31,32]). The RDE is essentially the opposite of the distance effect found in numerical comparison tasks: the closer the numerosities in a pair / triplet are in value, the quicker and better is one to judge whether these are in ascending order. The finding of RDEs for order judgement tasks, but not for numerical comparison tasks, suggests that different cognitive mechanisms are involved when processing ordinality and magnitude (e.g. [3,19,33–35]). In line with this, Goffin & Ansari [4] showed that the standard and RDEs are uncorrelated across participants. Thus, the RDE is thought to represent order-specific mental processes, which are qualitatively different from processes involved in magnitude judgements [36], and which may enable the rapid recognition of consecutively ordered items (e.g. [19,31,33,36]). Numerical order processing has also been shown to be a reliable predictor of arithmetic performance, both in adults (e.g. [3,4,37,38]) and in children (e.g. [39–41]).

Taken together, these studies highlight the importance of ordinality in the process of assigning meaning to numerical stimuli and the relationships symbols have to one another and thus challenge the proposal that the ANS is the sole underlying system which solves the symbol-grounding problem. However, these proposed solutions to the symbol-grounding problem need not be mutually exclusive. Although numerical symbols may be somewhat dissociated from the quantities they represent, it is still unclear whether ordinal or magnitude information is used to attach meaning to the symbols as both are usually concurrently present.

When attempting to differentiate the role(s) of magnitude and ordinal information in learning number symbols one crucial problem remains: symbolic numbers, i.e. Arabic digits, always convey both cardinal and ordinal meaning. Thus, when using comparison or ordering tasks with Arabic digits, it is difficult to differentiate between the two types and tell which one individuals use to solve a given task. We do not know how individuals can use magnitude and ordinal information to make different kinds of numerical judgement and the extent to which the characteristics of performance on numerical processing tasks (e.g. numerical distance effect (NDE) and RDE) are driven by either magnitude or ordinal information. Understanding this has important implications for modelling how symbolic numerical symbols are learned and how meaning might be attached to these symbols. Due to adults' high familiarity with both ordinality and magnitude, it is difficult to study the differential effects of these two sources of numerical information in adults when using familiar number symbols. Similarly, testing the individual contributions of ordinal and magnitude information to symbol learning in children is not straightforward because children receive a vast amount of both types of information when learning numbers. A different methodological approach is therefore needed.

### 1.1. Artificial symbol learning experiments

One approach to combat these problems is by using artificial symbol learning paradigms. These allow the comparison of contrasting instructional methods [25], and the complete control of all stimuli as well as how new symbols are learned and what type of information is provided. As learning takes place in a controlled environment, the amount of information received overall, but also for individual symbols, is controlled, not only within a participant but also across participants to ensure there are no differences.

In the numerical cognition domain, artificial symbol learning experiments have been previously used to explore different factors that may influence number symbol learning. For example, van den Berg *et al*. [42] presented neural data which suggested a possible distinction between mapping small (1–4) versus slightly larger (6–9) non-symbolic quantities onto novel symbols. Lyons & Beilock [43] investigated the effects of working memory (WM) on the ability to associate novel symbols with quantities and to learn their ordinal relations. They found that participants could infer ordinality from magnitude training and, furthermore, that both the ratio and WM capacity influenced performance on a symbolic comparison task that involved the symbols. The results showed that participants with higher WM go beyond symbol-quantity associations in the initial learning of the symbols and include symbol-symbol associations in their representations of the symbols, suggesting that ordinal knowledge about symbols facilitates the learning of the numerical meaning of those symbols and may influence how successfully these are learned initially [43]. In line with this, both Merkley & Scerif [44] and Lyons & Ansari [45] found that participants could infer the order of symbols from magnitude training and, because classical ratio effects were found, that participants processed the numerosities of the dot arrays, and thus the numerical meaning of the symbols, indicating the symbols were treated in a similar way to Arabic digits.

These studies show that artificial symbol learning paradigms are a useful technique to investigate and lend some evidence to show how adults can attach semantic or numerical meaning to, and infer ordinal relations of, abstract, novel symbols from magnitude training (e.g. [25,43–47]). However, most previous studies have focused on training with magnitude information alone. Therefore, we do not know how this compares to training with ordinal information or a combination of ordinal and magnitude information. Although Merkley *et al*. [25] compared how magnitude and order information influenced forming symbolic representations for artificial symbols, they found no difference in event-related potentials between an ordinal and magnitude learning group, suggesting similar formation of symbolic representations, and further highlighting the possible importance of ordinal information. Nevertheless, we still do not know whether the characteristics of performance on numerical processing tasks (i.e. ratio effects on magnitude comparison tasks and RDEs on ordinality tasks) are dependent on the involvement of magnitude or ordinal information, respectively.

### 1.2. The present studies

The current research used an artificial symbol learning paradigm to investigate learning novel symbols and the ability to map between these symbols and non-symbolic quantities. By using such a paradigm, we are able to specifically test the individual effects of both ordinal and magnitude information on the learning of novel numerical symbols. Our aim was to explore whether adults can learn the numerical meaning of seven novel symbols with ordinal, magnitude or a combination of ordinal and magnitude information. More specifically, Study 1 compared ordinal and magnitude training to investigate: (i) whether adults who received only magnitude information can learn the ordinal meaning of symbols and perform above chance on ordinality tasks, (ii) whether participants who received only ordinal information can infer magnitude meaning and perform above chance on magnitude comparison tasks, (iii) whether training with magnitude or ordinal information was more effective overall and (iv) whether training with magnitude or ordinal information is necessary for the presence of typical ratio and RDEs. In Study 2, we extended the design and findings of Study 1 to investigate the impact of training with a combination of both magnitude and ordinal information.

## 2. Study 1

### 2.1. Design and procedure

Before data collection, the study research questions, sample size, exclusion criteria and analysis plan were pre-registered. The preregistration is available at https://aspredicted.org/x6cq6.pdf.

A between-subjects design with two training groups was used. A convenience sample of 40 adults aged 18–48 (*M* = 24.42, s.d. = 6.77, 23 female) participated, providing 80% statistical power to detect a medium-effect size (based on a pilot study with a similar design) for the main between-groups effect in a 2 × 3 analysis of variance (ANOVA) design. Subjects received £3 as an inconvenience allowance. The experiment was completed on a 15.6″ laptop computer (resolution of 1920 × 1080 pixels) in a quiet laboratory and lasted approximately 20–25 min. All studies were approved by the Loughborough University Ethics Approvals (Human Participants) Sub-Committee and all participants gave written informed consent to take part.

Participants were randomly assigned to one of five different symbol sets so that the symbol order was counterbalanced across participants (i.e. sets consisted of the same symbols, but in different orders). Participants were randomly allocated to one of the two training groups, but all participants completed the post-training tasks in fixed order: symbolic comparison, order judgement, cross-modal comparison and global ordering (not computerized). Participants could take regular breaks throughout the experiment.

Artificial symbols were created using LaTeX. Symbols were centred on a white background and were 120 × 120 pixels in size. Dot arrays were created using Matlab. Each dot array contained black dots on a white background and was 350 × 350 pixels. No dots were overlapping and the number of dots per array was a multiple of six (i.e. 12, 18, 24, 30, 36, 42 and 48). For each quantity, 20 different dot arrays were created. The materials can be found at https://figshare.com/s/9d299ae5a2c1fa00795b.

### 2.2. Training phase

Both training groups used the same seven symbols and participants passively viewed each symbol 60 times.

**Figure 1.**
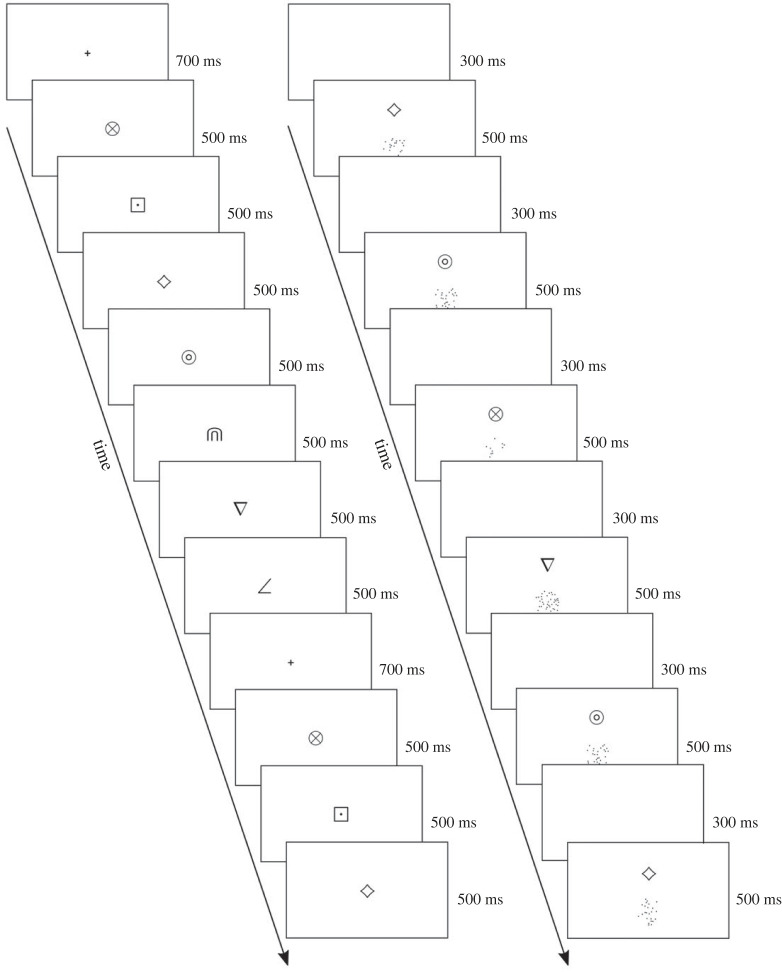
Example trials for the ordinal (left) and magnitude (right) training group in Study 1.

Participants in the ordinal training group were repeatedly presented with an ordered sequence of the symbols and were asked to ‘learn the order the symbols are presented in’. Symbols were presented one by one in the middle of the screen. Every sequence (i.e. seven symbols) was preceded by a fixation cross. Participants in the magnitude training group were presented with a symbol-dot pairing. Before each pairing, a white screen appeared. The task was to ‘learn which symbol goes with which approximate number of dots'. Trials were presented in a random order. The number of dots associated with a given symbol was counterbalanced across participants, but was fixed within a participant's experiment. The dot array on any trial was chosen randomly from the 20 versions available for that quantity. Figure 1 shows a schematic illustration of both training groups.

### 2.3. Test phase

Participants completed four tasks: symbolic comparison, order judgement, cross-modal comparison and global ordering (not computerized). For all computerized tasks, trials were presented until a response was given or a maximum of 3 s. The next trial was started once a response was given. No feedback was provided at any point.

During the symbolic comparison task, participants were simultaneously presented with two symbols appearing side by side. Participants were instructed to ‘indicate which one of them is larger. In other words, indicate which one represents more’. Every trial was preceded by a fixation cross (400 ms). If participants thought the symbol displayed on the left represented the higher quantity participants pressed the ‘z’ key, otherwise participants pressed the ‘m’ key. Each symbol was paired with every other symbol twice. The ratio on a given trial was determined by dividing the numerical value of the smaller symbol by the numerical value of the larger symbol. Ratios ranged from 0.25 to 0.875. Trials were ordered randomly. The display side, and thus the correct response key, was counterbalanced. Chance was at 50%. There were 168 trials.

During the order judgement task, participants were simultaneously presented with three symbols. Trials were ordered randomly. Participants were asked to ‘determine whether the symbols are in the correct ascending order’. If the symbols were in the correct order, participants pressed the ‘z’ key. If they were not in the correct order participants pressed the ‘m’ key. No descending orders were used. There were equal numbers of ascending and mixed-order trials. Within mixed-order trials, 50% were trials on which participants had to verify all three symbols before responding and 50% on which participant could respond by verifying only the first two symbols. The distance between the symbols was kept constant within a trial (i.e. only symmetric triplets were used) but varied across trials between one and three (e.g. distance 1: 1, 2, 3; distance 2: 1, 3, 5; distance 3: 1, 4, 7). There were 144 trials.

During the cross-modal comparison task, participants were simultaneously presented with one symbol and one dot array. Participants were asked to ‘indicate which one of them is numerically larger. In other words, indicate which one represents more’. If the left display represented the higher quantity, participants pressed the ‘z’ key, otherwise participants pressed the ’m’ key. The display sides and therefore the correct response keys were counterbalanced. Trials were ordered randomly. Each symbol was paired with two randomly selected dot arrays (of the 20 available versions for that quantity) corresponding to the numerical value of each of the other symbols. Thus, as in the symbolic comparison task, each symbol was compared to the numerical value of each of the other symbols. The ratios were the same as for the symbolic comparison task. There were 168 trials. Participants did not receive any calibration or indication of the range of numerosities used.

For the global ordering task, participants received seven laminated cards (approximately 6 × 6 cm) in random order, each containing one symbol. Participants were verbally instructed to lay the cards on the table so that all the symbols were in the correct ascending order from left to right, starting with the smallest symbol.

### 2.4. Results

We first present the pre-registered analyses, including pre-processing, descriptive statistics and inferential statistics. The main analyses involve separate one-sample *t*-tests to test if each group performed above chance on each task. 2 × 3 ANOVAs are performed to test for group differences in accuracy and RT across the computerized tasks and 2 × 2 ANOVAs are used to test for distance effects. Significant main effects and interactions are investigated further with *post hoc* Bonferroni-corrected *t*-tests. Separate by-item regressions for each task and group are used to test for ratio effects.

Second, we present exploratory analyses, which were not pre-registered. All analyses were conducted using JASP (JASP Team, 2018). The data analysis files can be found at https://figshare.com/s/555d3faf3a31e3a99f48.

Trials using the smallest or largest symbol of the sequence were excluded from all analyses. We anticipated that participants could use these as anchor points, which could aid solving the tasks but may also inflate accuracy rates. We omitted the smallest and largest symbols because we wanted to see the true effect of our training conditions on the learning of both the order (especially for the magnitude group) and the magnitude (for the ordinal group) without the influence of endpoints. We believe that the exclusion of the endpoints better allows us to identify whether participants could learn the meaning of the symbols beyond the use of these anchors^1^. For all studies, the main variables of interest were mean accuracy and median RTs. When RT data are used, we include correct trials only. If the Shapiro–Wilk test indicated a violation of normality, Wilcoxon signed-rank tests were carried out on the accuracy data instead of one-sample *t*-tests. Because we analysed both accuracies and RTs, the alpha level was set as 0.025 in order to correct for multiple comparisons.

### 2.5. Pre-registered analyses

#### 2.5.1. Groups’ performance on the tasks

**Figure 2.**
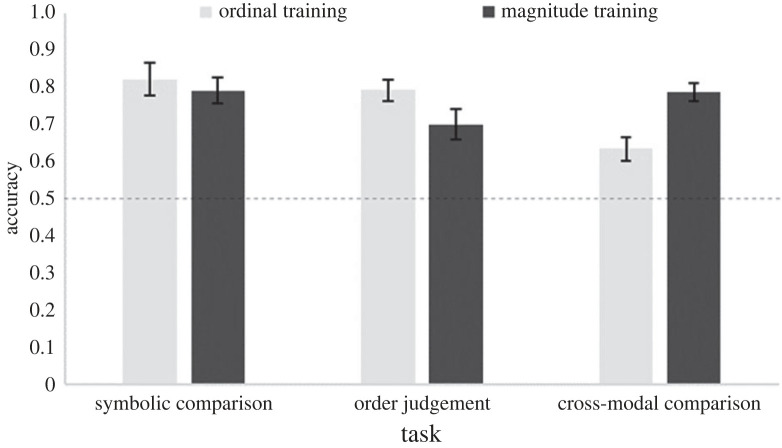
Accuracy rates for each training group for the three computerized test tasks in Study 1. Error bars indicate ± 1 s.e. of the mean. The dashed line indicates chance level.

**Figure 3.**
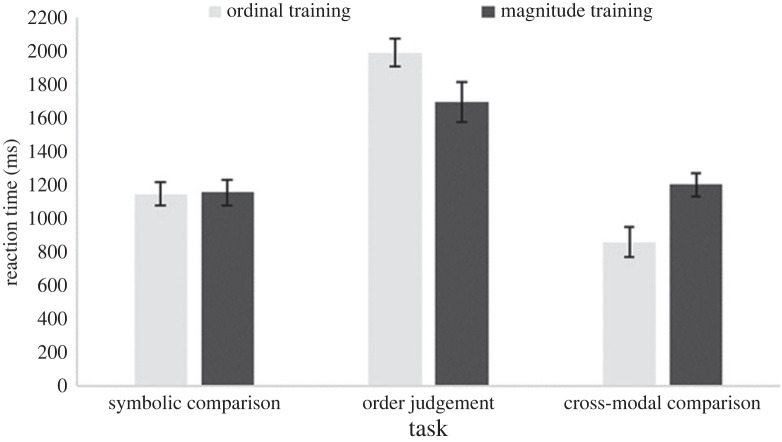
RTs for each training group for the three computerized tasks in Study 1. Error bars indicate ± 1 s.e. of the mean.

Figures 2 and 3 show the accuracies and RTs for each group for the three computerized test tasks, respectively. The ordinal training group was significantly above chance on all tasks (all *ps* < 0.001). The magnitude group was also significantly above chance on all tasks (all *ps* < 0.001).

To test for a difference between training groups on the computerized tasks, we conducted a 2 (training group: ordinal; magnitude) × 3 (tasks: symbolic comparison; order judgement; cross-modal comparison) mixed ANOVA. Using accuracy data, a significant main effect of task, *F*_2,76_ = 7.38, *p* = *0*.001, $\eta_{p}^{2}=0.16,$ and a significant task × training group interaction, *F*_2,76_ = 12.68, *p* < *0*.001, $\eta_{p}^{2}=0.25,$ were found, but no significant main effect of training group, *F* < 1. Independent samples *t*-tests revealed no significant group differences on the symbolic comparison, *t*_38_ = 0.52, *p* = 0.603, or order judgement task, *t*_38_ = 1.88, *p* = 0.067. Welch's *t*-test revealed that, as we expected from the design of our study, the magnitude group (*M* = 0.79, s.d. = 0.11) performed significantly better than the ordinal group (*M* = 0.63, s.d. = 0.14) on the cross-modal task, *t*_35.79_ = 3.91, *p* < 0.001, *d* = 1.24. Using RT data revealed a significant main effect of task, *F*_2,76_ = 85.59, *p* < 0.001, $\eta_{p}^{2}=0.69,$ and a significant task×training group interaction, *F*_2,76_ = 11.27, *p* < 0.001, $\eta_{p}^{2}=0.23.$ There was no significant main effect of training group, *F* < 1. *Post hoc* independent samples *t*-tests on the interaction revealed a significant difference in RTs on the cross-modal comparison task, *t*_38_ = 2.95, *p* = 0.005, *d* = 0.93, whereby the ordinal training group (*M* = 860.26, s.d. = 407.95) was significantly faster than the magnitude training group (*M* = 1202.10, s.d. = 320.75). There was no significant group difference on the symbolic comparison, *t*_38_ = 0.08, *p* = *0*.939, or the order judgement task, *t*_38_ = 1.99, *p* = 0.054.

#### 2.5.2. Distance effects

During the order judgement task, trials could either be in the correct ascending order or out of order. To investigate the presence of distance effects on this task, a 2 (distance: 1; 2) × 2 (order: in order; out of order) repeated measures ANOVA, with training group as a between-subjects factor was conducted. As trials including the smallest or largest symbol were excluded from all analyses, no trials with a distance of 3 between the symbols remained. Therefore, only trials with distances of 1 and 2 were analysed. Using accuracy data showed a significant effect of distance, *F*_1,38_ = 15.94, *p* < 0.001, $\eta_{p}^{2}=0.30,$ but no other significant main effects or interactions (all *ps* > 0.046). A paired samples *t*-test revealed that participants performed better with greater distance, *t*_39_ = 3.84, *p* < 0.001, *d* = 0.61. Contrary to the expected RDE, these results indicate a standard distance effect across both in order and out of order trials. Analysis of RT data showed no significant main effects or interactions (all *ps* > 0.09).

#### 2.5.3. Ratio effects

**Figure 4.**
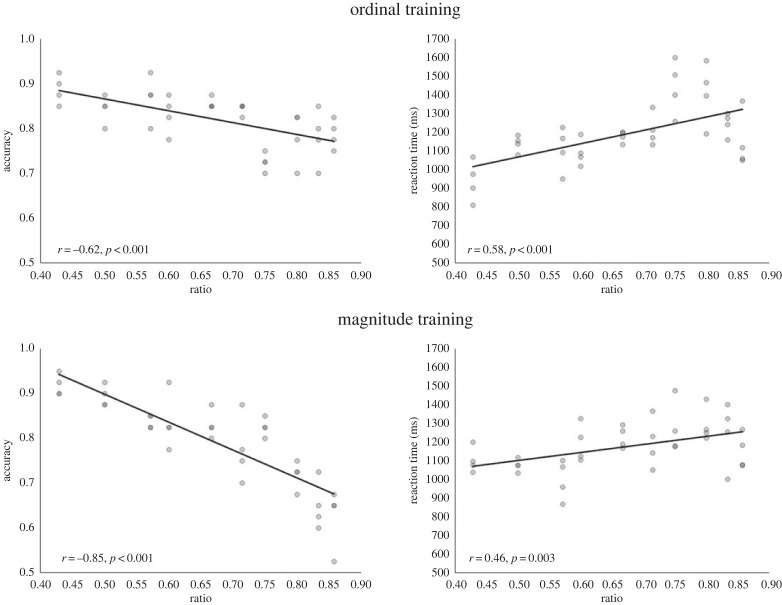
Correlations between accuracy and ratio and RT and ratio for each training group for the symbolic comparison task in Study 1.

To test for ratio effects on the comparison tasks and compare these across training groups, by-items regressions with three factors (training group, ratio and ratio×training group interaction) were conducted. The ordinal training group served as a baseline and thus its group value was set to zero, whereas the magnitude training group was assigned a value of one. For the symbolic comparison task, analysis of accuracy data revealed that training group, *β* = 1.26, *t*_76_ = 3.70, *p* < 0.001, ratio, *β* = −0.43, *t*_76_ = −4.50, *p* < 0.001, and the interaction effect, *β* = −1.50, *t*_76_ = −4.30, *p* < 0.001, were significant predictors, $R_{\mathrm{adj}}^{2}=0.63,$ *F*_3,76_ = 46.20, *p* < 0.001. Correlational analyses (figure 4) revealed ratio was significantly negatively correlated with accuracy for both the ordinal, *r*_38_ = −0.62, *p* < *0*.001, and the magnitude training group, *r*_38_ = −0.85, *p* < 0.001, on the symbolic comparison task. Thus, ratio affected accuracy of both training groups, but more so the magnitude group compared to the ordinal training group. The analysis of RT data revealed that ratio, *β* = 0.65, *t*_76_ = 4.79, *p* < 0.001, was the only significant predictor of RT, $R_{\mathrm{adj}}^{2}=0.27,$ *F*_3,76_ = 10.52, *p* < 0.001 and thus ratio affected RT for both groups similarly.

**Figure 5.**
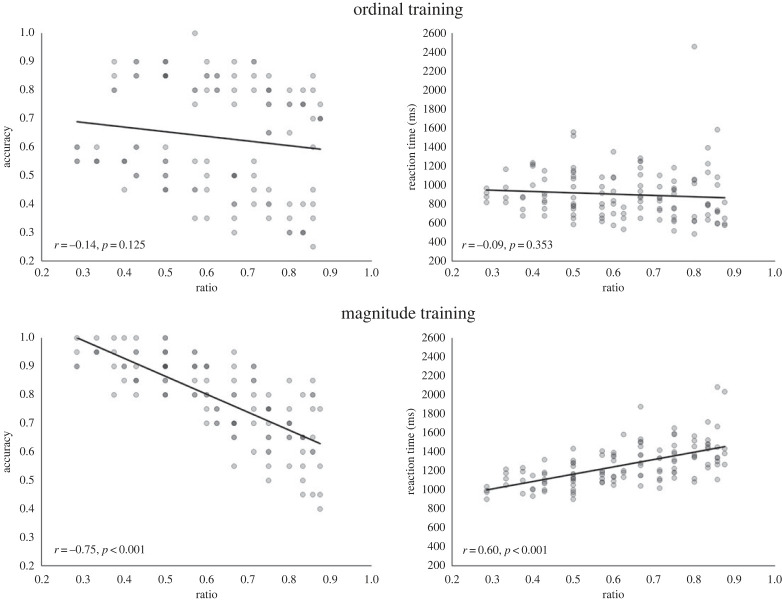
Correlations between accuracy and ratio and RT and ratio for each training group for the cross-modal comparison task in Study 1.

For the cross-modal task, the analysis of accuracy data revealed that training group, *β* = 1.20, *t*_236_ = 5.88, *p* < 0.001, and the ratio × training group interaction, *β* = −0.84, *t*_236_ = −4.00, *p* < 0.001, but not ratio itself, *β* = −0.15, *t*_236_ = −1.96, *p* = 0.051, were significant predictors, $R_{\mathrm{adj}}^{2}=0.33,$ *F*_3,236_ = 40.60, *p* < 0.001). With RT data, only the ratio×training group interaction, *β* = 1.00, *t*_236_ = 5.20, *p* < 0.001, was a significant predictor, $R_{\mathrm{adj}}^{2}=0.44,$ *F*_3,236_ = 63.40, *p* < 0.001. The significant interaction effects indicate that the ratio affected the training groups differently. Correlational analyses (figure 5) confirmed that ratio had very little effect on the RT or accuracy for the ordinal training group (RT: *r*_118_ = −0.09, *p* = 0.353; accuracy: *r*_118_ = −0.14, *p* = 0.125), but significantly affected the magnitude group (RT: *r*_118_ = 0.60, *p* < 0.001; accuracy: *r*_118_ = −0.75, *p* < 0.001).

### 2.6. Exploratory analyses

#### 2.6.1. Symbol position effects

**Figure 6.**
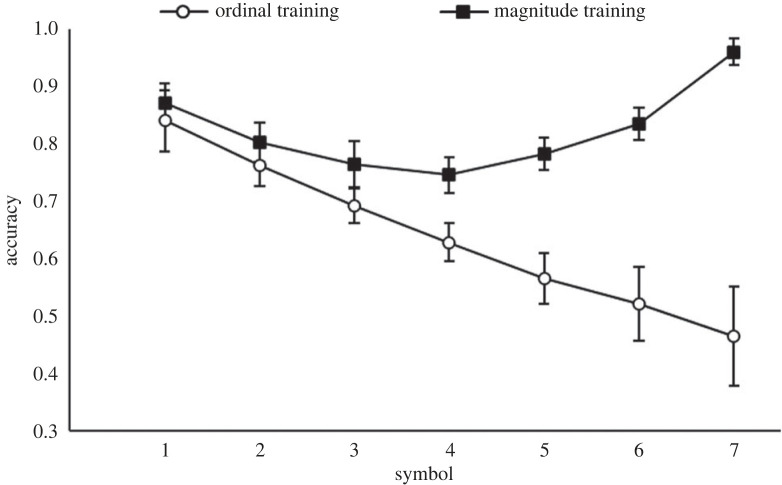
Accuracy for the ordinal and magnitude training group on each symbol for the cross-modal comparison task in Study 1. Trials including the smallest and largest symbols were included to show performance across the full sequence. Error bars indicate ± 1 s.e. of the mean.

Due to a lack of ratio effects for the ordinal training group, we investigated whether the specific position of a symbol in the sequence affected accuracy on the cross-modal task. As with the above analyses, we excluded trials including the smallest and largest symbols. After correcting the degrees of freedom using Greenhouse–Geisser estimates of sphericity (ɛ = 0.59), a 2 (training group) × 5 (symbol position) mixed ANOVA on accuracy showed a significant main effect of training group, *F*_1,38_ = 15.25, *p* < 0.001, $\eta_{p}^{2}=0.29,$ and symbol position, *F*_2.36, 89.74_ = 4.75, *p* = 0.008, $\eta_{p}^{2}=0.11,$ and a significant training group×symbol position interaction, *F*_2.36, 89.74_ = 7.23, *p* < 0.001, $\eta_{p}^{2}=0.16.$ Visual inspection of the interaction graph (figure 6) showed very different patterns for the two groups: for the magnitude group, performance was highest for trials including the second (80% correct) and sixth (83.5% correct) symbols, but performance dropped for the middle symbols. By contrast, performance for the ordinal training group declined as symbols came later in the sequence and represented higher quantities. Accuracy declined from 76.2% for symbol 2 to 52.1% for symbol 6.

#### 2.6.2. Block analysis

**Figure 7.**
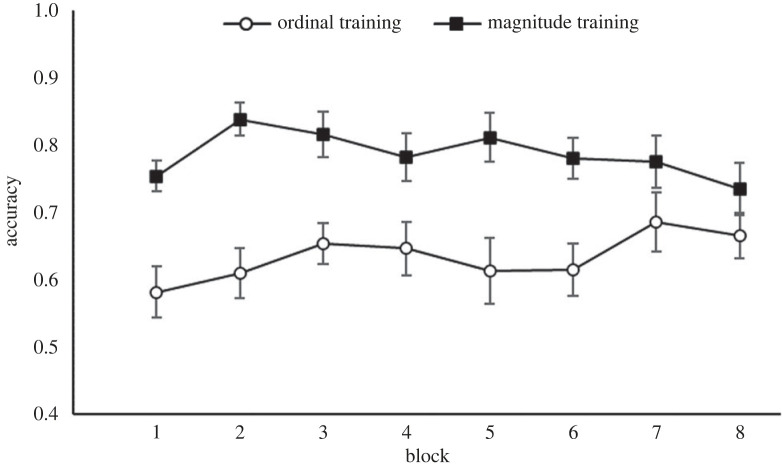
Accuracy rates of the magnitude and ordinal training group on each block of trials of the cross-modal comparison task. Trials including the smallest and largest symbols were excluded. Error bars indicate ± 1 s.e. of the mean.

Since the ordinal training group received no magnitude information during the training phase, we investigated if performance on the first block of trials of the cross-modal comparison task was worse than that of all other blocks of trials, as this was the first time participants in the ordinal training group encountered dot arrays. To compare performance across all blocks and between groups, a 2 (training group: ordinal; magnitude) × 8 (blocks of trials) mixed ANOVA was conducted. This revealed only a main effect of group, *F*_1,38_ = 15.63, *p* < 0.001, $\eta_{p}^{2}=0.29,$ but no significant effect of block, *F*_7,266_ = 1.45, *p* = 0.184, and no interaction, *F*_7,266_ = 2.18, *p* = 0.037. Figure 7 shows accuracy rates for both groups for each block of trials. A one-samples *t*-test on the accuracy of the first block of the ordinal training group showed that this group did not perform significantly above chance on the first block, *t*_19_ = 2.15, *p* = 0.044, *d* = 0.48. The same analysis for the magnitude training group showed that this group was significantly above chance on the first block, *t*_19_ = 11.15, *p* < 0.001, *d* = 2.49.

#### 2.6.3. Global ordering task

Lastly, participants' ability to re-construct the global order was tested. Accuracy was calculated in terms of whether a symbol was placed in its correct position in the sequence. If a symbol was in its correct position, a score of 1 was given, otherwise a score of 0 was given. For example, if the correct sequence was 1 234 567—whereby each value represents one specific symbol—and a participant's order was 1324657, then a score of 3 would be given, leading to an accuracy of 0.429. Chance level for this task is 14.29%. Two Wilcoxon signed-rank tests revealed that both the ordinal training, *W* = 210, *p* < 0.001, and the magnitude training group, *W* = 200, *p* < 0.001, were significantly above chance. Welch's *t*-test indicated that the ordinal training group (*M* = 0.94, s.d. = 0.14) was significantly better than the magnitude training group (*M* = 0.62, s.d. = 0.35), *t*_24.60_ = 3.77, *p* < 0.001, *d* = 1.19.

### 2.7. Discussion

Our main aim was to investigate whether both ordinal- and magnitude-based judgements can be made on the basis of either magnitude or ordinal information alone. The results of this study indicated that both ordinal and magnitude training allows adults to compare and order novel symbols, but also to compare these to non-symbolic representations of magnitude. Furthermore, we found that performance of the magnitude training group on the symbolic comparison and cross-modal comparison tasks was more strongly affected by the ratio than the ordinal training group, indicating the groups solved these tasks in different ways. This conclusion is also supported by the symbol position effects and overall group differences on the cross-modal comparison task. The magnitude training group was likely to have used the absolute magnitude information from the training phase to make magnitude judgements about the symbols. By contrast, the ordinal training group can only have inferred relative magnitude information from the ordinal training. Just like knowing absolute magnitudes, knowledge about the relative magnitudes of the symbols can also be directly applied to the symbolic comparison task, explaining the lack of group differences in this task. Relative magnitude information cannot be directly applied and used on the cross-modal task. Nevertheless, it appears that with only minimal information derived from the trials of the task itself, participants in the ordinal training group were able to combine this information with the relative magnitude information learned during the training phase, allowing them to successfully complete the task. This conclusion is further supported by the significant group differences and symbol position effects we found.

A combination of magnitude and ordinal information is, in fact, one of the proposals for how children learn symbol meaning. Carey [10,48,49] suggested that symbolic numbers gain meaning through combining the initial knowledge of small exact magnitudes with the increasing knowledge of ordinal relationships. Study 1 did not include a training group who were provided with both ordinal and magnitude information during training. Therefore, Study 2 extended the findings of Study 1, by using ordinal training and a combination of ordinal and magnitude training (denoted here as ordinal + magnitude training) to investigate the effect of the combination of these two types of information. Specifically, we investigated whether relatively small amounts of magnitude information for some of the symbols, in combination with ordinal information for all symbols, would affect performance on a cross-modal comparison task compared to the learning group which only received ordinal information about the symbols. Given that participants in Study 1 who received ordinal training were nevertheless able to make cross-modal comparison judgements, we expected that providing some absolute magnitude information during training would improve performance. However, it remains to be tested whether this improvement would extend across the entire range of symbols learned (as implied by Carey's theory) or only apply to the symbols for which participants received the additional magnitude information.

## 3. Study 2

### 3.1. Design and procedure

Before data collection, the study research questions, sample size, exclusion criteria and analysis plan were pre-registered. The preregistration is available at https://aspredicted.org/cm7n5.pdf.

A between-subjects design with two training groups was used. A convenience sample of 60 adults aged 19–61 (*M* = 27.83, s.d. = 8.03, 31 female) participated, providing 80% statistical power to detect a large main effect in an independent samples *t*-test. Subjects received £3 as an inconvenience allowance. The experiment was completed on a laptop computer and lasted approximately 15 min.

The symbols were the same as in Study 1. Dot arrays were created using Python. For each quantity, 20 different dot arrays were created. All dot arrays were on a white background and were 200 × 200 pixels in size. The materials can be found at https://figshare.com/s/57f04b6e56e57bb1c9fa.

Participants were randomly allocated to one of five symbol sets and to one of two training groups. Following the training, all participants completed all test tasks in order: a cross-modal comparison and a global ordering task. Participants could take regular breaks throughout the experiment.

### 3.2. Training phase

Both training groups used the same seven symbols. The training was split into ten blocks. The ordinal training group was the same as in Study 1 and each of the 10 training blocks contained the sequence six times, thus each symbol was seen 60 times (420 total exposures).

**Figure 8.**
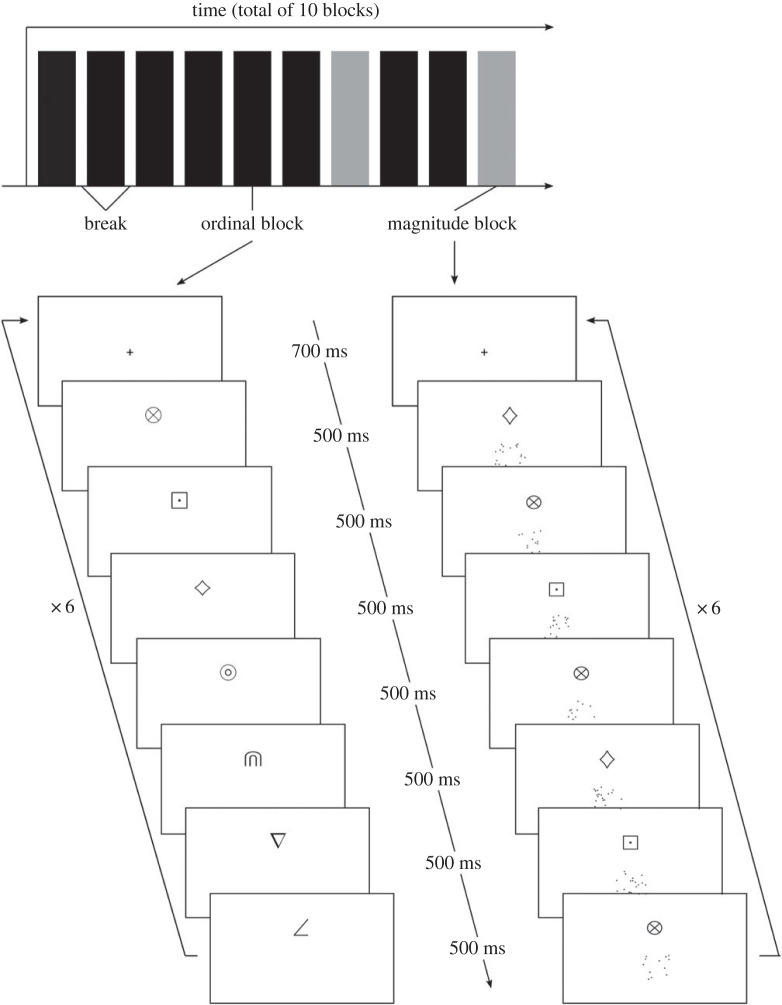
Example schematic illustration of the training phase of the ordinal + magnitude training group in Study 2. The order of the eight ordinal (black bars) and two magnitude blocks (grey bars) was randomized.

In the ordinal + magnitude training group, participants were presented with the sequence of the symbols (ordinal information) as well as pairings of a symbol and a dot array (magnitude information). Magnitude information was given for the first three symbols only. The symbol was always presented at the top and the dot array at the bottom of the screen. Of the 10 training blocks, eight provided ordinal information and two provided magnitude information. The order of blocks was random. A given block contained either six repetitions of the symbol sequence or 42 symbol-dot pairings. This means symbols 1, 2 and 3 were seen 76 times each and symbols 4–7 were seen 48 times each (420 total exposures). Figure 8 gives a schematic illustration. The dot array that was paired with a corresponding symbol on any magnitude information trial was chosen randomly from the 20 versions available for that quantity.

### 3.3. Test phase

The cross-modal comparison task was similar to that in Study 1. However, each symbol was compared to dot arrays representing six different quantities. Three quantities were smaller than the symbol (approximate ratios: 0.67; 0.75; 0.83) and three were larger than the symbol (approximate ratios: 1.17; 1.33; 1.5). Each individual trial was repeated between two and four times resulting in 224 trials. This was done to ensure that if participants based their answer on a congruency-based strategy, that is on comparing the dot array to the approximate average of the range of dot arrays included in the experiment, then this would lead to chance performance (50%).

The global ordering task was the same as in Study 1.

### 3.4. Results

As in Study 1, we first present the pre-registered analyses. Our main analyses involve one-sample *t*-tests to test for above-chance performance on each task as well as on the full trial set and the subset. Independent sample *t*-tests are used to compare the groups' accuracy and RT across the tasks. 2 × 2 ANOVAs are used to compare each group's performance on trials where both received the same and different information. By-item regressions are again used to test for ratio effects. Second, we present exploratory analyses.

Trials using the first or last symbol of the sequence were excluded from all analyses. The data analysis files can be found at https://figshare.com/s/91d22756c0fd104314a6.

### 3.5. Pre-registered analyses

#### 3.5.1. Groups’ performance on the tasks

**Figure 9.**
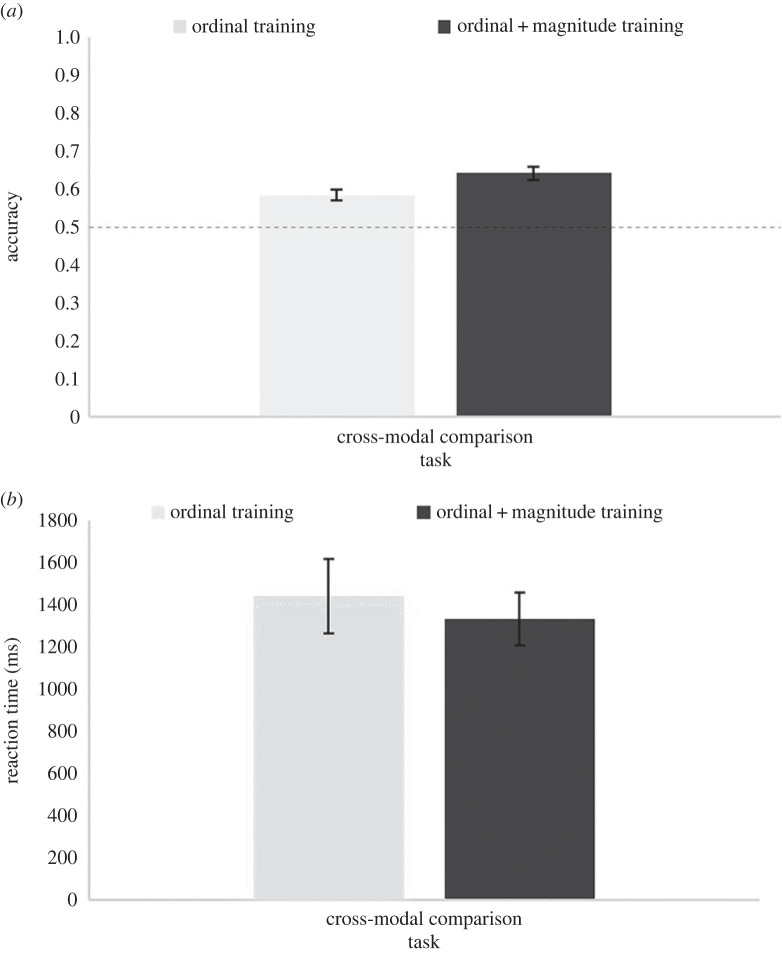
Accuracy rates (top) and RTs (bottom) for each training group for the cross-modal comparison task in Study 2. Error bars indicate ± 1 s.e. of the mean. The dashed line indicates chance level.

Figure 9 shows the accuracies and RTs for each training group on the cross-modal comparison task. Analyses revealed that both the ordinal and the ordinal + magnitude training group were significantly above chance on the cross-modal task (both *ps* < 0.001). An independent samples *t*-test revealed a significant accuracy difference between the two training groups, *t*_58_ = 2.75, *p* = 0.008, *d* = 0.71, with accuracy lower for the ordinal training group (*M* = 0.58, s.d. = 0.08) than the ordinal + magnitude training group (*M* = 0.64, s.d. = 0.09). No differences were found on the RT data, *t*_58_ = 0.50, *p* = 0.617.

To test whether both training groups were above chance on the global ordering task, Wilcoxon signed-rank tests were carried out. Both groups were significantly above chance (all *ps* < 0.001). Welch's *t*-test showed the ordinal training group (*M* = 0.88, s.d. = 0.18) performed significantly better than the ordinal + magnitude training group (*M* = 0.65, s.d. = 0.38), *t*_42.00_ = 3.04, *p* = 0.004, *d* = 0.78.

**Figure 10.**
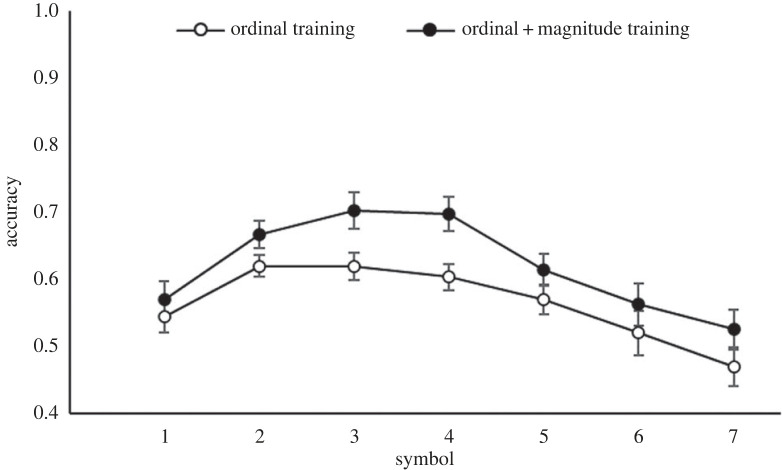
Accuracy for the ordinal and ordinal + magnitude training group on each symbol for the cross-modal comparison task in Study 2. Trials including the smallest and largest symbols were included to show performance across the full sequence. Error bars indicate ± 1 s.e. of the mean.

During the training phase, both training groups received ordinal information, while the ordinal + magnitude training group received additional magnitude information for the first three symbols. Thus, it was of interest to see whether there was a difference in performance between the two training groups on those trials where both groups received the same information (i.e. only ordinal; trials using symbols 4, 5, 6) and on trials where they received different information (i.e. where the ordinal + magnitude training group received additional information; trials using symbols 2, 3). A 2 (training group) × 2 (symbols: 2, 3; 4, 5, 6) mixed ANOVA on accuracy data showed a main effect of symbol, *F*_1,58_ = 9.37, *p* = 0.003, $\eta_{p}^{2}=0.14,$ and of training group, *F*_1,58_ = 8.61, *p* = 0.005, $\eta_{p}^{2}=0.13,$ but no interaction, *F* < 1. Notably, this lack of an interaction indicates that the additional information about symbols 2 and 3 provided to the ordinal + magnitude training group was successfully used to improve performance on the other symbols, about which no additional magnitude information was provided (figure 10). Using RT data revealed no main effect of symbol, *F*_1,58_ = 2.17, *p* = 0.146, or training group, *F* < 1, and no interaction, *F* < 1.

#### 3.5.2. Ratio effects

As in Study 1, we investigated whether the ratio of each trial affected performance. A by-items regression with three factors (training group, ratio and ratio × training group interaction) was conducted on the accuracy data. This revealed an overall significant model, *F*_3,116_ = 5.08, *p* = 0.002, $R_{\mathrm{adj}}^{2}=0.09,$ but neither the training group, *β* = 0.87, *t*_116_ = 0.96, *p* = 0.338, nor the ratio itself, *β* = −0.20, *t*_116_ = −1.58, *p* = 0.117, nor the interaction, *β* = −0.66, *t*_116_ = −0.73, *p* = 0.467, were significant predictors. Using RT as the outcome variable showed an overall non-significant model, *F*_3,116_ = 1.88, *p* = 0.136, with no significant predictors. The by-item regressions were repeated for a subset of trials where both training groups received only ordinal information (trials using symbols 4, 5, 6). Using accuracy, results showed an overall non-significant model, *F*_3,68_ = 1.22, *p* = 0.308, with no unique predictors. Using RT yielded the same results, *F*_3,68_ = 1.42, *p* = 0.244.^2^

### 3.6. Exploratory analyses

#### 3.6.1. Symbol position effects

As in Study 1, a 2 (training group) × 5 (symbols) ANOVA investigated whether the symbol position in the sequence influenced performance. As the assumption of sphericity was violated, *χ*^2^ (9, *N* = 60) = 48.17, *p* < *0*.001, degrees of freedom were corrected using Greenhouse–Geisser estimates of sphericity (ɛ = 0.65). There was a significant main effect of training group, *F*_1,58_ = 8.38, *p* = 0.005, $\eta_{p}^{2}=0.13,$ and of symbol, *F*_2.62, 151.68_ = 10.66, *p* < 0.001, $\eta_{p}^{2}=0.16,$ but no interaction, *F* < 1. Figure 10 shows the accuracy by symbol for each training group. Crucially, this shows that the ordinal + magnitude training advantage extended beyond the magnitude-trained symbols (1–3) to the ordinal-only-trained symbols (4–7).

#### 3.6.2. Block analysis

**Figure 11.**
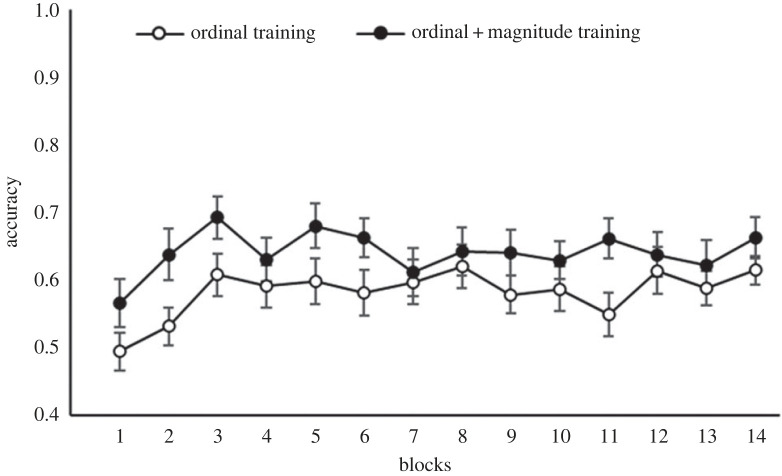
Accuracy rates of the ordinal and ordinal + magnitude training group on each block of trials of the cross-modal comparison task in Study 2. Trials including the smallest and largest symbols were excluded. Error bars indicate ± 1 s.e. of the mean.

To explore whether the different training groups led to levels of performance that varied over the blocks of the experiment, we conducted a 2 (training group: ordinal; ordinal + magnitude) × 14 (blocks of trials) mixed ANOVA. This revealed a main effect of group, *F*_1,58_ = 7.19, *p* = *0*.010, $\eta_{p}^{2}=0.11,$ and of block, *F*_13,754_ = 2.05, *p* = 0.015, $\eta_{p}^{2}=0.03,$ but no significant interaction, *F* < 1. Figure 11 shows accuracy rates for both groups for each block of trials. A one-sample *t*-test on the accuracy of the first block of trials for the ordinal training group revealed that they did not perform significantly above chance on this block, *t*_29_ = −0.22, *p* = 0.825. Repeating this analysis for the ordinal + magnitude training group shows that this group was also not above chance on the first block of trials, *t*_29_ = 1.82, *p* = 0.080.

### 3.7. Discussion

Participants in the ordinal + magnitude training group were not only better able to compare the symbols to non-symbolic quantities than the ordinal training group, but they also extrapolated the absolute magnitude meaning of a subset of the symbols to all other symbols. This study therefore replicated and extended the results of Study 1.

## 4. General discussion

Across two experiments, we have shown that individuals can learn the meaning of, and make relatively accurate numerical judgements about, artificial number symbols after having received either ordinal- or magnitude-based training. We first consider what this allows us to infer about how participants use different types of numerical information before discussing the implications for theories which address the symbol-grounding problem.

### 4.1. Inferring magnitude and order

With our studies, we sought to uncover the impact of ordinal and magnitude training regimes on participants' performance on numerical processing tasks and what the effects observed on these tasks indicated about how participants used the different types of numerical information when processing symbols. Study 1 revealed that participants who received magnitude information could infer the ordinal structure and relations of the symbols based on the magnitude information provided during training. This replicates previous studies of artificial symbol learning which have demonstrated that participants can learn symbols from magnitude information [43,44]. Yet most of these studies have not investigated the impact of ordinal training. Our results showed that participants could accurately compare and order novel symbols after receiving either ordinal or magnitude training. We found that participants who received magnitude information (Study 1) or ordinal information with additional magnitude information for a subset of the symbols (Study 2) could compare the novel symbols to non-symbolic representations more accurately than participants who received only ordinal training. Interestingly, although the ordinal group did not perform significantly above chance on the first block of trials on the cross-modal symbol comparison task, as we would have expected, but they did so from the second block onwards, suggesting that they can make magnitude judgements based on the magnitude information in the test task despite not having received magnitude information during the training phase. We discuss this in more detail below. Nevertheless, receiving only ordinal information appears to be sufficient to infer the magnitude of the symbols and succeed in our cross-notation task.

While Merkley *et al*. [25] also compared ordinal and magnitude training, though their main analyses were focused on electrophysiological data, they used a symbolic comparison task and did not employ a cross-notation task. Our results from the symbolic comparison task in Study 1, however, corroborate those of Merkley *et al*. as they also found no group differences and concluded that ‘adults can form symbolic representations based on either numerical magnitude or order information alone’ [25, p. 286]. Nevertheless, they did not investigate whether a combination of ordinal and magnitude training could further facilitate learning. Our Study 2 revealed that additional magnitude information may indeed be beneficial for learning and expanding the numerical meaning of artificial number symbols. These results analogously support a recent study [50] showing that cardinality understanding helps children to expand their understanding of ordinality beyond the count-list, but importantly helps combine the concepts of ordinality and cardinality. The results from these previous studies together with our results provide further evidence for the important role that both ordinal and cardinal information play in the acquisition of the semantic meaning of numerical symbols or symbolic number knowledge more broadly.

### 4.2. How participants used the information provided in the training phase

#### 4.2.1. Ratio effects

Although we have shown that both ordinal and magnitude training can be used to learn the numerical meaning of and make numerical judgements about novel artificial symbols, we still do not know exactly how participants used the information provided in the training to solve the tasks. Ratio effects, which are commonly found on magnitude comparison tasks with either non-symbolic dot arrays or Arabic digits (e.g. [51,52]), have often been taken to indicate that a numeral activates its underlying magnitude representation [19] and thus been commonly used to support the proposal that the ANS is involved in symbolic number processing. Thus, if ratio effects indicate underlying magnitude activation, then we would expect ratio effects for all training groups on the cross-modal comparison task, as the task itself involved absolute non-symbolic magnitudes, which participants had to actively process in order to compare them to a symbol. Our results, however, suggest that the presence or absence of ratio effects is more complex and that one needs to be cautious about drawing strong inferences from the presence or absence of these effects.

For the magnitude training group, it is at least plausible that magnitude representations were activated in the various comparison tasks, as participants were trained to associate novel symbols with approximate, but absolute magnitudes. The ordinal + magnitude training group received some absolute magnitude information for a subset of symbols, and our results suggest that this additional information allowed participants to extrapolate this magnitude information to all the symbols, leading to improved performance when comparing novel symbols to non-symbolic quantities. Nevertheless, since participants in the ordinal training group had been given no magnitude information at all, it is hard to see how they could have activated any underlying magnitude representations when completing our comparison task, at least not directly. Thus, if ratio effects indicate underlying magnitude activation, then a lack of ratio effects should indicate a lack of magnitude processing.

Both groups showed ratio effects on the symbolic comparison task, however, the ordinal group in Study 1 did not show this effect on the cross-modal comparison task, nor did we find ratio effects for either training group in Study 2 on this task. Thus, we found a lack of ratio effects on a task where we would have expected to find these (i.e. cross-modal comparison task), and we found ratio effects on a task where we would not have necessarily expected these (i.e. symbolic comparison task). This therefore raises the question of what can be concluded from the experimental observation of ratio effects. That is, if an experimenter observes a ratio effect, is it legitimate to infer that participants activated underlying magnitude representations, in a similar manner to how dot arrays activate ANS representations? In other words, if ratio effects indicate magnitude activation, then why did participants in our ordinal training group show this effect on our symbolic comparison task? This task does not require any knowledge of absolute magnitudes and can be solved by comparing the symbols based on their relative ordinal positions in the symbol sequence. It seems plausible then that the ratio effects found for the ordinal group stem from comparing the symbols based on their symbol-symbol associations, and those found for the magnitude group stem from magnitude-based comparisons. Indeed, previous research has suggested that ratio effects, and thus also size and distance effects, can also be caused by the semantic relations between symbols (e.g. [26,53–55]). Our results therefore suggest that the presence of ratio effects does not necessarily imply that magnitude processing took place.

Given this and the lack of ratio effects for the ordinal groups (and the ordinal + magnitude group), we therefore need to further explore how the training groups solved the tasks, especially how the ordinal group solved the cross-modal comparison task, and what type of information they may have used.

#### 4.2.2. Relative order versus relative magnitude

The lack of ratio effects for the ordinal group on the cross-modal, but not the symbolic comparison task suggests that they are likely to have used different processes to solve these two tasks. With regard to the cross-modal comparison task, there are two reasonable hypotheses that explain how the ordinal group may have tackled this task. One possibility is that the ordinal group inferred the relative magnitude of the symbols in the sequence during training and then, when faced with early trials of the cross-modal task, and being exposed to the range of magnitudes involved, were able to convert this relative magnitude information into absolute magnitude information to solve the task. Alternatively, the ordinal group may have instead inferred order from the magnitudes used in the task and made comparisons based on the relative ordinal positions, e.g. 5th largest symbol versus 3rd greatest dot quantity.

We believe that our results favour the first hypothesis for two reasons. First, the second hypothesis seems most plausible when the comparison task involves a restricted number of magnitudes. Further, we would expect that the number of magnitudes involved would have an impact on performance, i.e. decreasing performance with increasing number of magnitudes because the range of magnitudes as well as the variety of ratios is increased. Although the ordinal training in both of our studies was identical, the cross-modal comparison task differed in its set-up. Specifically, in Study 1, there were a total of seven different magnitudes used in the task, i.e. each symbol was compared only to the six magnitudes represented by each of the other symbols. In Study 2, however, we employed a total of 29 different magnitudes. Each symbol was still compared to six magnitudes, but these were based on pre-determined ratios and not only on the magnitudes of the other symbols. The reason for this change was to provide a more rigorous test of how well participants had learned the symbols at the beginning and end of the sequence. If the second hypothesis were correct, we would expect that the ordinal group would perform worse in Study 2 compared to Study 1 because they would need to infer ordinal information from 29 magnitudes instead of 7. We examined our data for this and found no statistically significant difference between the two ordinal training groups across the studies (*t*_26.641_ = 1.479, *p* = 0.151).

Second, considering performance on the initial blocks of the cross-modal task, it seems likely that the ordinal training group was only able to successfully complete this task after they had experienced some absolute magnitude information from the task itself and therefore gained a sense of the range of magnitudes involved in the study, which could then be combined with the previously learned ordinal information. Indeed, previous studies have demonstrated that only small amounts of magnitude information are required to shift the associations between numerical symbols and magnitudes [56]. We saw a similar effect here: in Study 1, the ordinal training group was not able to succeed on the first block of trials of the cross-modal task. Similarly, neither the ordinal nor the ordinal + magnitude group in Study 2 was able to succeed on the first block of trials, yet the ordinal + magnitude group succeeded from the second block onwards, but the purely ordinal group still did not succeed on that block. This shows that the ordinal groups in both studies needed time to encounter some of the range of magnitudes in the task, (and that they needed more time (i.e. more trials) in Study 2 because the range of magnitudes was greater), before they could successfully solve the task. Once they had a sense of the range, they could apply the relative magnitude information they had acquired through training and can then complete the task. This process happened more quickly for participants in the combined training group who had received a subset of this information during training. Taking together, we favour the first hypothesis that participants who received (mainly) ordinal information about the artificial symbols solved the cross-modal comparison task by inferring relative magnitude from the ordinal information they were provided with, coupled with the range of magnitudes shown in the cross-modal task.

#### 4.2.3. (Reverse) distance effects

Both ordinal and magnitude training groups showed a standard NDE on the order judgement task, i.e. performance was higher for trials with larger numerical distances. Typically, RDE are observed for order judgement tasks, where performance is highest for trials with small numerical distances (e.g. [36,57]). This effect has been interpreted to stem from stronger associations between numbers that are closer (e.g. 2–3 – 4) than those that are further apart (e.g. 2–4 – 6) [33,36] and/or the role of verbal count sequence knowledge [31,58]. The fact that we found no evidence of an RDE and instead evidence of an NDE for the order judgement task suggests that the processes involved in completing the artificial symbol order judgement task are not the same as those involved in an Arabic numeral order judgement task. An NDE is typically observed when participants complete magnitude comparison tasks and therefore it is possible that participants here relied on sequential comparison processes rather than holistic order judgements. Participants in this study had no verbal labels for the symbols (i.e. had no verbal count sequence information) and therefore count sequence information could not give rise to an RDE.

#### 4.2.4. The role of additional magnitude information

In Study 2, we incorporated small amounts of additional magnitude information for a subset of symbols into the training to directly compare performance of a purely ordinal training group to an ordinal training group with this additional absolute information. As discussed above, results from the cross-modal comparison task indicated that participants who received additional absolute magnitude information were better able to compare symbols to non-symbolic quantities than participants without this information. Furthermore, participants extrapolated the numerical meaning from the subset of symbols across the whole set of symbols as the numerical representations held for these symbols were calibrated. Thus, the finding of significant group differences and symbol position effects confirms the beneficial effect of having at least some additional absolute magnitude information available when learning the numerical meaning of artificial symbols. However, this additional magnitude information was not as beneficial as additional exposure to the order information training when participants were tested on their ability to recreate the global order of the sequence. The global order task did not require numerical judgements *per se*, and it is evident that when required to simply learn a sequence, the most effective training is to maximize exposure to that sequence.

### 4.3. Implications for the symbol-grounding problem

How do these results fit with the existing literature, and what do they mean for the symbol-grounding problem? We believe our results can be used to model and give insights into the type of information that may be involved in the learning of numerical symbols. Various solutions to the symbol-grounding problem have been proposed, for example that symbols are mapped onto non-symbolic quantities (the ANS mapping account), but also that symbols gain meaning through their relations to other symbols (the symbolic estrangement hypothesis).

Furthermore, Carey [10,48,49] proposed that the combination of increasing knowledge of ordinal relationships and the initial magnitude mappings onto small, subitizable magnitudes gives rise to the meaning of symbolic numbers. Although our magnitudes were approximate and outside of the subitizing range, our results demonstrated that having small amounts of additional absolute magnitude information for an initial subset of symbols during the training phase improved the ability to learn the numerical meaning of and make numerical judgements about all symbols. As Study 1 showed that participants attached meaning to the symbols from both ordinal and magnitude information alone, it is not surprising that combining these types of information in Study 2 also allowed meaning to be attached. Here, for the ordinal + magnitude training group, magnitude information was given in addition to the ordinal information, but only for the first three symbols of the sequence. Thus, participants learned the initial magnitude meaning for only the first few symbols through the training. Next, they appeared to extract this magnitude meaning of the symbol subset, combined this information with their knowledge about the ordinal relations between all the symbols, and then generalized this to the rest of the symbols so that magnitude meaning could be inferred for the entire set of symbols. We believe that this process is analogous to that proposed by Carey's theoretical model. Had we found that adults are unable to learn the meaning of the symbols and complete the comparison tasks, we believe that this would pose a problem to Carey's theory because if adults are unable to do this, it is unlikely that children can. Although the present studies did not directly test this proposal, our results provide some indirect support for this model as adults in our studies were able to attach meaning to the symbols and complete numerical tasks with these, but more importantly they extrapolated the magnitude meaning from a subset of symbols and apply this to the whole range of symbols. We believe that it is this generalizability aspect—being able to use the type of information provided for the initial symbols of the sequence to apply and generalize this to the remaining symbols of the sequence—that is analogous to Carey's theory, rather than testing the processes by which meaning might be attached.

The present work only compared a purely ordinal training group to either a purely magnitude group or a combined information group, thus no conclusions can be drawn about potential differences between a purely magnitude group and a combined information group and thus about the amount of magnitude information that is needed to improve the learning of the numerical meaning of the symbols. Future studies could incorporate a greater variety of learning groups into one study to directly compare multiple different instructional methods. Furthermore, it would be valuable to further investigate the role of ordinal and magnitude information, and the mapping between them, in the context of larger as well as smaller quantities, perhaps also covering quantities in the subitizing range. Lastly, it would also be beneficial to compare participants' general mapping ability between Arabic digits and non-symbolic quantities to their ability to map between novel artificial symbols and non-symbolic quantities, in order to get a better understanding of the factors underlying the learning of artificial number symbols.

## 5. Conclusion

In conclusion, we provide evidence that, with relatively little exposure, adults can infer the ordinal and magnitude meanings of artificial number symbols from either ordinal, magnitude or a combined training. We further demonstrate that after training participants can also make accurate judgements about, and map between, these symbols and non-symbolic quantities. Small amounts of magnitude information for a subset of symbols—in addition to ordinal information about the whole range of symbols—allowed the extrapolation of the magnitude meaning, and the application of this to the whole set of symbols.

## Data Availability

The materials used for Experiment 1 can be found at https://doi.org/10.17028/rd.lboro.13645847. This link can be found in the ‘Design and Procedure’ Section of Study 1 in the manuscript. Materials contain the stimuli, dot arrays for each quantity (in .bmp format) and five sets of symbol orders (in.png format), as well as two PsychoPy files, one for each training group, and the associated Excel condition files needed to run the experiment in PsychoPy. The data files for Experiment 1 can be found at: https://doi.org/10.17028/rd.lboro.13645832. This link can be found in the ‘Results’ Section of Study 1 in the manuscript. Under this link can be found (i) the data analysis files (JASP files) and the .csv files needed to run the JASP files, (ii) .html exports of the results from the JASP files and (iii) the raw data files, one for each of the three tasks (symbolic comparison, order judgement, cross-modal comparison). ‘ReadMe’ text documents are provided to guide the reader and to describe the content of the other files. The materials used for Experiment 2 can be found at https://doi.org/10.17028/rd.lboro.13645853. This link can be found in the ‘Design and Procedure’ Section of Study 2 in the manuscript. Materials contain the stimuli, dot arrays for each quantity (in .png format) and five sets of symbol orders (in .png format), as well as one PsychoPy file and the associated Excel condition files needed to run the experiment in PsychoPy. The data files for Experiment 2 can be found at: https://doi.org/10.17028/rd.lboro.13645850. This link can be found in the ‘Results’ Section of Study 2 in the manuscript. Under this link can be found (i) the data analysis files (JASP files) and the .csv files needed to run the JASP files, (ii) .html exports of the results from the JASP files and (iii) the raw data file for the cross-modal comparison task. ‘ReadMe’ text documents are provided to guide the reader and to describe the content of the other files.

## Footnotes

1 We have also run the full set of analyses with the smallest and largest symbol of the sequence included in the data. The results remain unchanged.

2 We also pre-registered an analysis designed to test whether participants used a strategy where decisions about the magnitude of the dot arrays were based on the median of the range of numerosities of all dot arrays. One-sample *t*-tests indicated that participants were not significantly above chance on trials where the strategy leads to the incorrect answer, suggesting participants may have made some use of this strategy. This is unsurprising as the ordinal training group needed to use some strategy to solve the task in the absence of magnitude calibration during training.
